# Supplementary material for: Double cross-linked 3D layered PBI proton exchange membranes for stable fuel cell performance above 200 °C
Source: Nat Commun. 2024 Apr 22;15:3409. doi: 10.1038/s41467-024-47627-4 (PMC11035571; doi:10.1038/s41467-024-47627-4)
Supplement: Supplementary file 1 — Supplementary Information [file 41467_2024_47627_MOESM1_ESM.pdf]

Supplementary Materials for

Double Cross-linked 3D Layered PBI Proton Exchange Membranes for Stable Fuel Cell Performance Above 200°C

Liang Zhang[1], Mengjiao Liu[1], Danyi Zhu[1], Mingyuan Tang[1], Taizhong Zhu[1], Congjie Gao[1], Fei Huang[1]* and Lixin Xue[2,3]*

[1]Center for Membrane Separation and Water Science & Technology, College of Chemical Engineering, Zhejiang University of Technology, Hangzhou, Zhejiang, 310014, China.

[2]College of Chemistry and Materials Engineering, Wenzhou University, Wenzhou, Zhejiang, 325035, China.

[3]Institute of New Materials & Industrial Technologies, Wenzhou University, Wenzhou 325024, China.

*Corresponding author. E-mail: feihuang@zjut.edu.cn; xuelx@zjut.edu.cn

Supplementary Figure 1. Mechanical properties of membranes. (a) Creep compliance of PA-doped DC-PBI-G with varied branched monomer contents. (b) Creep compliance of PA-doped m-PBI-D, p-PBI-G, HO-PBI-G and DC-PBI-G. (c) Mechanical integrity and recoverability of PA-doped and undoped DC-PBI-G membranes after twisting and folding.

Supplementary Figure 2. SEM images of PA-undoped DC-PBI-G-240 membrane. (a) Surface view and (b) cross-sectional view.

Supplementary Figure 3. WAXD spectra of undoped DC-PBI-G-240.

Supplementary Figure 4. Solid-state ^{31}P NMR spectra of PA-undoped membranes. (a) DC-PBI-G and (b) DC-PBI-G-240, with external reference to $(NH_4)_2HPO_4$ (solid).

Supplementary Figure 5. Radial distribution function of DC-PBI/PPA and DC-PBI/PA.

Supplementary Figure 6. Microstructure of gel-state PBI membranes. (a) 2D SAXS images and (b) SAXS patterns of the undoped DC-PBI-G membrane in both in-plane and through-plane directions. Quantitative analysis includes (c) layer thickness, (d) inter-layer spacing, and (e) diameter of nano-filaments, derived from SEM images.

Supplementary Figure 7. MD simulations. 3D spatial configuration and 2D local enlarged views of DC-PBI molecular chains (cubic box size: 12×12×12 nm^3) in (a) PPA, (b) PA, and (c) after PA removal.

Supplementary Figure 8. FT-IR spectra of various PA-undoped membranes.

Supplementary Figure 9. Membrane synthesis. (a) Synthetic reactions and (b) photographs of PA-doped DC-PBI-G, DC-PBI-G-240, HO-PBI-G, and p-PBI-G membranes. The manufacturing of membranes was scaled up to about 10 cm × 35 cm.

Supplementary Figure 10. TGA curves of PA-undoped PBI membranes.

Supplementary Figure 11. Changes in ADL over time at 240 °C. ADLT and ADLA for PA-doped (a) DC-PBI-G, (b) p-PBI-G. All error bars indicate the s.d. of at least three independent measurements.

Supplementary Figure 12. PA retention capability at 80 °C/40% RH. (a) The ADL values and (b) retention of PA-doped membranes. All error bars indicate the s.d. of at least three independent measurements.

Supplementary Figure 13. Comparative proton conduction in two directions. (a) Schematic diagram of proton transport pathways along the in-plane and through-plane directions. (b) Comparison of in-plane and through-plane proton conductivity for DC-PBI-G. (c) Comparison of in-plane and through-plane proton conductivity for p-PBI-G. All error bars indicate the s.d. of at least three independent measurements.

Supplementary Figure 14. Through-plane proton conductivity of PA-doped DC-PBI-G-240 and p-PBI-G-240 membranes at varied temperatures. All error bars indicate the s.d. of at least three independent measurements.

Supplementary Figure 15. Intrinsic through-plane proton conductivity comparison of PA-undoped DC-PBI-G-240 and m-PBI-D-240 membranes. All error bars indicate the s.d. of at least three independent measurements.

Supplementary Figure 16. H_2 crossover of PA-doped PBI membranes. (a) Equivalent H_2 crossover current densities of the fuel cell based on PA-PBI membrane as a function of operating temperature with ambient pressure at 0.5 V. (b) Linear voltammetry sweeps for Nafion® 211[1] at 80 °C, PBI[2] at 160 °C, and DC-PBI-G at both 80 °C and 160 °C.

Supplementary Figure 17. Single-cell performance of PA-doped DC-PBI-G under anhydrous conditions with dry H_2/O_2 at atmospheric pressure (Pt loading of 0.5 mg cm^{-2} on both sides).

Supplementary Figure 18. Single-cell performance of PA-doped DC-PBI-G-240 under anhydrous conditions with dry H_2/O_2 at atmospheric pressure and 240 °C (Pt loading of 0.5 mg cm^{-2} on both sides).

Supplementary Figure 19. High frequency resistance (HFR) of single cell based on PA-doped DC-PBI-G, p-PBI-G and m-PBI-D membranes at varied operating temperatures.

Supplementary Figure 20. Single cell performance of PA-doped DC-PBI-G membrane using dry methanol reformate gas (75% H_2, 24% CO_2 and 1% CO) and O_2 at atmospheric pressure without additional humidification. (a) Polarization curves and power density at varied operating temperatures. (b) Long-term durability at 0.2 A cm^{-2} and 220 °C.

Supplementary Figure 21. Single cell performance of PA-doped p-PBI-G and DC-PBI-G membranes at 160 °C using dry H_2 and air at atmospheric pressure without additional humidification (Pt loading of 1.0 mg cm^{-2} on both sides).

Supplementary Figure 22. Photographs of MEAs. The PA-doped (a) DC-PBI-G and (b) p-PBI-G based MEAs after a long-term durability test at 0.2 A cm^{-2} and 220 °C with dry H_2/O_2 under atmospheric pressure (Pt loading of 1.0 mg cm^{-2} on both sides).

Supplementary Figure 23. Low-temperature single cell performance of PA-doped DC-PBI-G membranes with dry H_2 and O_2 at atmospheric pressure without additional humidification (Pt loading of 1.0 mg cm^{-2} on both sides).

Supplementary Figure 24. Low-temperature FC durability and AST cycling tests. (a) Long-term durability of PA-doped DC-PBI-G MEA with H_2/O_2 at 0.2 A cm^{-2} and 40 °C. (b) Peak power density values of the PA-doped DC-PBI-G MEAs after the shut-down/start-up AST cycling at 40 °C. Testing conditions: Pt loading of 1.0 mg cm^{-2} on both sides, dry H_2/O_2 flow, without backpressure.

Supplementary Figure 25. Proton conductivity measurement. (a) Schematic of the two-probe conductivity testing setup. (b) Equivalent circuit model. (c) A typical Nyquist plot used for membrane ohmic resistance fitting. (d) Through-plane proton conductivity schematic diagram of the custom-designed impedance test cell utilized in this work.

**Supplementary Figure 26.**	Compression creep measurement. (a) Equations for creep compliance estimation. (b) Compression creep testing methodology, Maxwell model and experimental setup.
**Supplementary Table 1.**	Porosity and surface area of undoped PBI membranes.
**Supplementary Table 2.**	Comparison of WAXD peak position, area ratio and d-spacing.
**Supplementary Table 3.**	The elemental mass concentration of PA-undoped DC-PBI-G and p-PBI-G under high-temperature and KOH treatments.
**Supplementary Table 4.**	The relative integrated peak intensities of the ^{31}P NMR spectra.
**Supplementary Table 5.**	High-temperature creep compliance and ADL retention of membranes.
**Supplementary Table 6.**	H_2 crossover current densities and crossover rates of the fuel cell based on PA-PBI membrane as a function of operating temperature with ambient pressure at 0.5 V.
**Supplementary Table 7.**	Summary and comparison of representative LT-PEMs and their corresponding FC performance using H_2.
**Supplementary Table 8.**	Summary and comparison of representative HT-PEMs and their corresponding FC performance using H_2.
**Supplementary Table 9.**	Summary and comparison of the FC performance of representative HT-PEMs using reformate gas.
**References**	(1-32)

Polymer Synthesis and Membrane Preparation

HO-PBI-G Membrane. The gel-state HO-PBI-G membrane was prepared via a PPA sol-gel process. 3,3'-diaminobenzidine (TAB, 1.2724g) and 2,5-dihydroxyterephthalic acid (DHTA, 1.1766g) were dissolved in polyphosphoric acid (PPA, 120 g), then the reaction mixture was continuously stirred and purged with dry nitrogen in a 250 ml three-necked flask equipped with mechanical stirring. The reaction terminated 190 °C for 25 hours when a moderate viscosity was attained in the polymer solution. The resulting solution was then uniformly cast onto a glass substrate using a doctor blade, forming a 243 μm thick gel-state PA-doped HO-PBI-G membrane through sol-gel phase inversion under ambient temperature and 55%RH conditions for 24 hours.

p-PBI-G Membrane. The gel-state p-PBI-G membrane was prepared via a PPA sol-gel process. 3,3'-diaminobenzidine (TAB, 2.0692g) and terephthalic acid (TPA, 1.6043g) were dissolved in polyphosphoric acid (PPA, 120 g), then the reaction mixture was continuously stirred and purged with dry nitrogen in a 250 ml three-necked flask equipped with mechanical stirring. The reaction terminated 190 °C for 30 hours when a moderate viscosity was attained in the polymer solution. The resulting solution was then uniformly cast onto a glass substrate using a doctor blade, forming a 210 μm thick gel-state PA-doped HO-PBI-G membrane through sol-gel phase inversion under ambient temperature and 55%RH conditions for 24 hours.

m-PBI-D Membrane. The PA-doped m-PBI-D membrane was obtained by immersing the commercial m-PBI film, supplied by PBI Performance company, in an 85 wt% PA solution at 25 °C for 120 hours until a constant weight was achieved.

Characterizations

Scanning Electron Microscopy (SEM). Scanning Electron Microscopy (SEM) and Energy-Dispersive X-ray Spectrometry (EDX) were employed to investigate the micromorphology and elemental distribution of PA-undoped PBI membranes. The analysis was performed using a SEM-EDX (SU-8010, Hitachi, Tokyo, Japan) at an

accelerating voltage of 10 kV. Before SEM imaging, the samples were secured in place using conductive adhesive and subjected to a 90-second sputter-coating process. To prepare the samples for imaging, all the PA-doped membrane samples were initially immersed in water to remove PA, freeze-dried for 24 hours, and then fractured using liquid nitrogen.

Wide-angle X-ray Diffraction (WAXD). WAXD spectra of PBIs in undoped dry film form after freeze-drying were acquired employing a Rigaku X-ray diffractometer (R-AXIS RAPID) equipped with Cu **K*α*** radiation. The d-spacing (d_{sp}) was calculated utilizing Bragg's Equation, as outlined below:

$$d_{sp} = \frac{n \cdot \lambda}{2 \cdot sin\theta} \quad (1)$$

where n signifies an integral number (l, 2, 3), λ represents the X-ray wavelength, d_{sp} denotes the distance between polymer segments, and θ is the diffraction angle.

Small-angle X-ray Scattering (SAXS). SAXS patterns and 2D SAXS images of PBIs in undoped dry film form after freeze-drying were acquired using a Xeuss 2.0 equipped with Cu **K*α*** radiation in the q range of 0-0.3 $Å^{-1}$.

Compression Creep. Time-dependent creep behavior was assessed using a compression mechanical analyzer[3]. In a standard procedure, PA-doped membranes, with a 10 mm diameter, were shaped into cylindrical samples measuring 3 mm in thickness. These samples were pre-treated in a 180 °C oven for 24 hours. Subsequently, a constant 20% strain was applied to the samples, and force changes were monitored over 7 hours at a constant temperature of 180 °C, in order to simulate a typical HT-PEMFC operating condition. The resulting time-dependent stress data was used to evaluate creep compliance and creep rate. Each sample underwent three repeated compression creep tests, with an associated experimental error range of approximately 7%. Further details can be found in Supplementary Fig. 26.

ADL Retention under High-temperature Compression Mode. Cylindrical samples, comprised of 10 mm diameter PA-doped membranes stacked to a 3 mm thickness, were pre-treated for 24 hours at 180 °C. Subsequently, these samples were

exposed to a 240 °C environment with a constant 0.08 MPa stress for 30 minutes to replicate fuel cell operating conditions above 200 °C. Following the test, the membranes were wiped to eliminate leached PA, and ADLs in both the initial and remaining membranes were quantified via acid-base titration. The calculation of ADL retention was depicted in the following equation:

$$ADL\ retention\ \% = \frac{ADL_t}{ADL_0} \cdot 100 \quad (2)$$

where ADL_0 is the initial ADL of PA-doped membrane, ADL_t is the residual ADL after high-temperature compression.

Thermal Stability. Thermogravimetric analysis (TGA) of the prepared PBI membrane was conducted using a TGA Cahn TherMax 700 analyzer under N_2 atmosphere. The analysis was carried out in the temperature range of 50 to 900 °C at a heating rate of 10 °C min^{-1}.

Proton Conductivity Stability and Retention. The proton conductivity stability of PA-doped membranes was assessed at 240 °C under 0% RH, with measurements of membrane resistance taken every 30 minutes. Before initiating the tests, the PA-doped membrane samples underwent preheating at 120 °C for a duration of 5 hours to remove any residual moisture. The changes in through-plane proton conductivity (σ_t) were monitored. The retention of proton conductivity was determined using the following equation:

$$Conductivity\ Retention\ \% = \frac{\sigma_t}{\sigma_0} \cdot 100 \qquad (3)$$

where σ_0 is the initial proton conductivity after preheating.

Membrane Porosity. To determine the porosity, the PA-doped PBI membranes underwent PA removal process and were then immersed in deionized water at 25°C for 100 hours, resulting in a wet mass (M_{wet}) and a wet volume (V_{wet}). Subsequently, they were dried at 100 °C for 12 hours, yielding a dry mass (M_{dry}). The porosity was calculated using the following equation:

$$Porosity\ \% = \frac{M_{\mathrm{wet}} - M_{\mathrm{dry}}}{V_{\mathrm{wet}} \cdot \rho_{water}} \cdot 100 \quad (4)$$

where M_{wet} and V_{wet} represent the mass and volume of the membrane after immersion in deionized water, M_{dry} corresponds to the mass of the dried membrane, and ρ_{water} denotes the density of water.

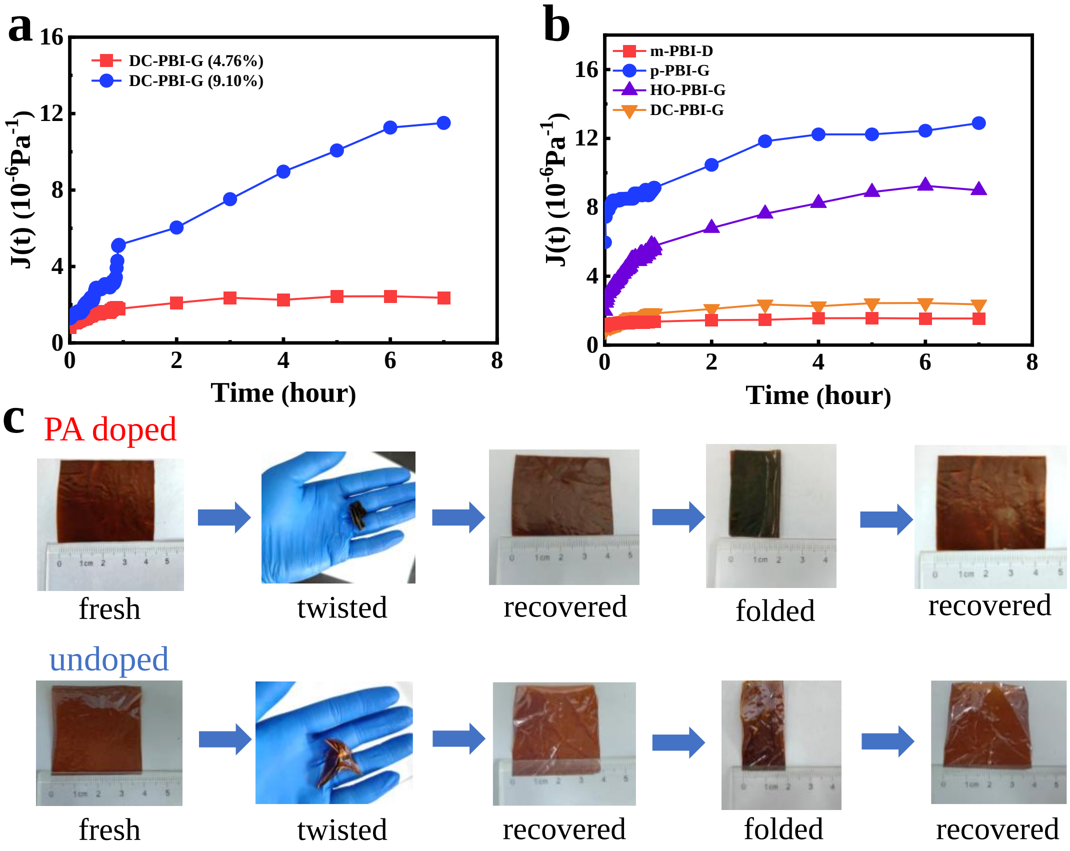

Supplementary Figure 1. Mechanical properties of membranes. (a) Creep compliance of PA-doped DC-PBI-G with varied branched monomer contents. (b) Creep compliance of PA-doped m-PBI-D, p-PBI-G, HO-PBI-G and DC-PBI-G. (c) Mechanical integrity and recoverability of PA-doped and undoped DC-PBI-G membranes after twisting and folding.

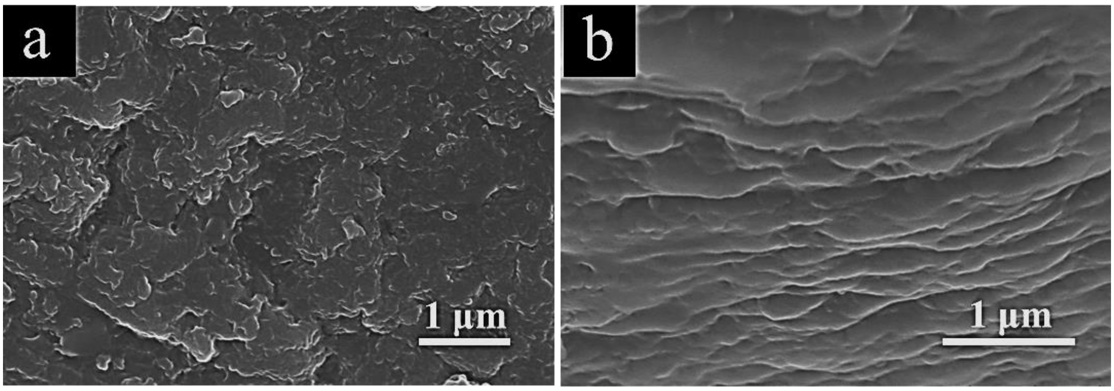

Supplementary Figure 2. SEM images of PA-undoped DC-PBI-G-240 membrane. (a) Surface view and (b) cross-sectional view.

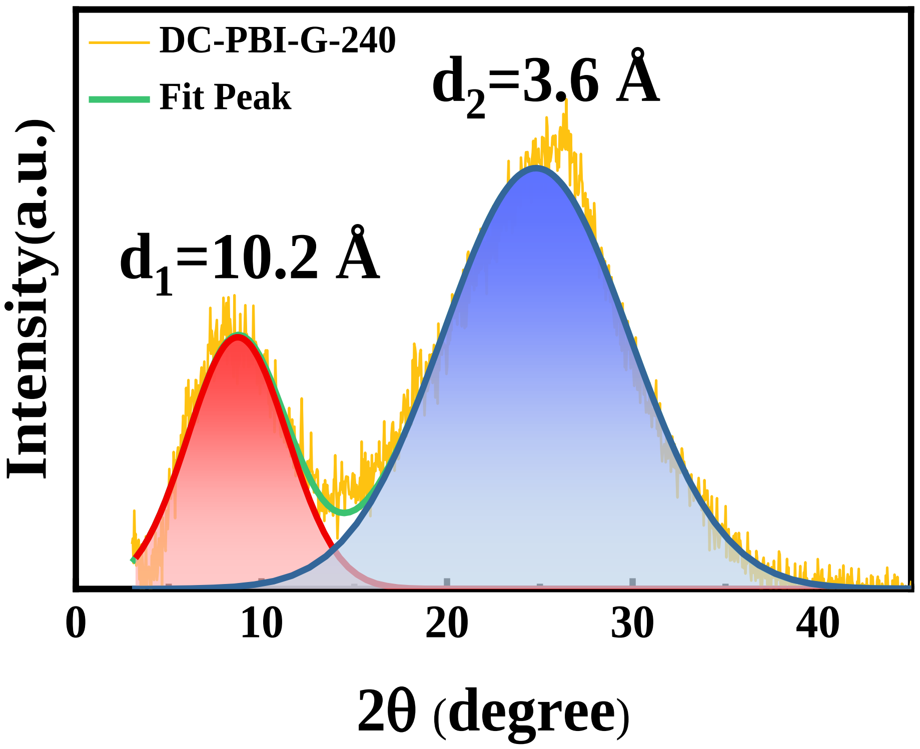

Supplementary Figure 3. WAXD spectra of undoped DC-PBI-G-240.

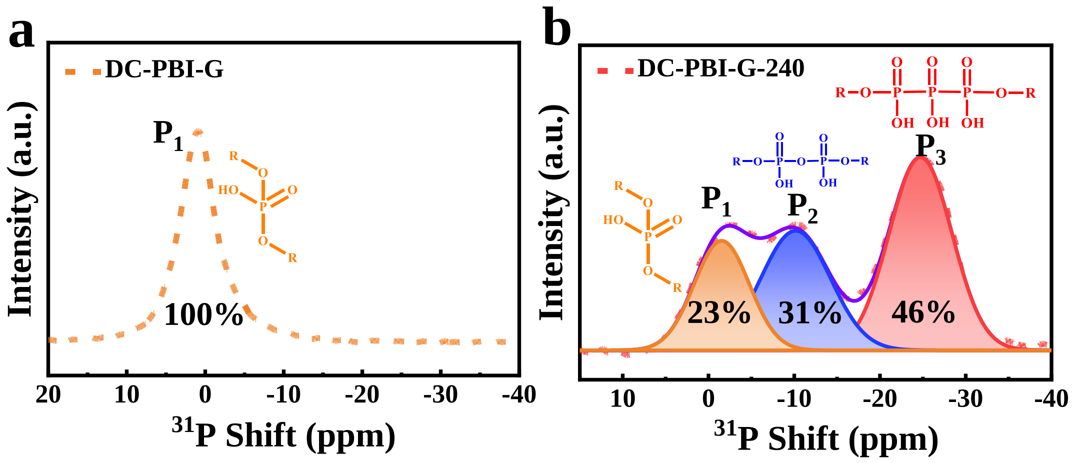

Supplementary Figure 4. Solid-state ^{31}P NMR spectra of PA-undoped membranes. (a) DC-PBI-G and (b) DC-PBI-G-240, with external reference to $(NH_4)_2HPO_4$ (solid).

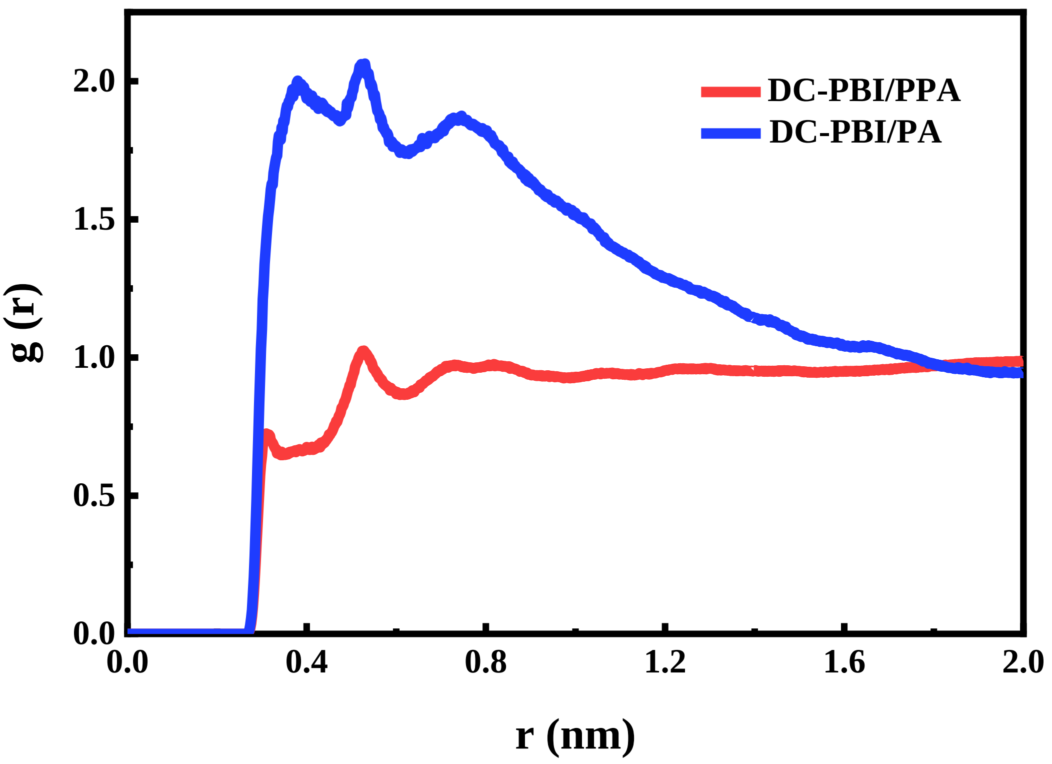

Supplementary Figure 5. Radial distribution function of DC-PBI/PPA and DC-PBI/PA.

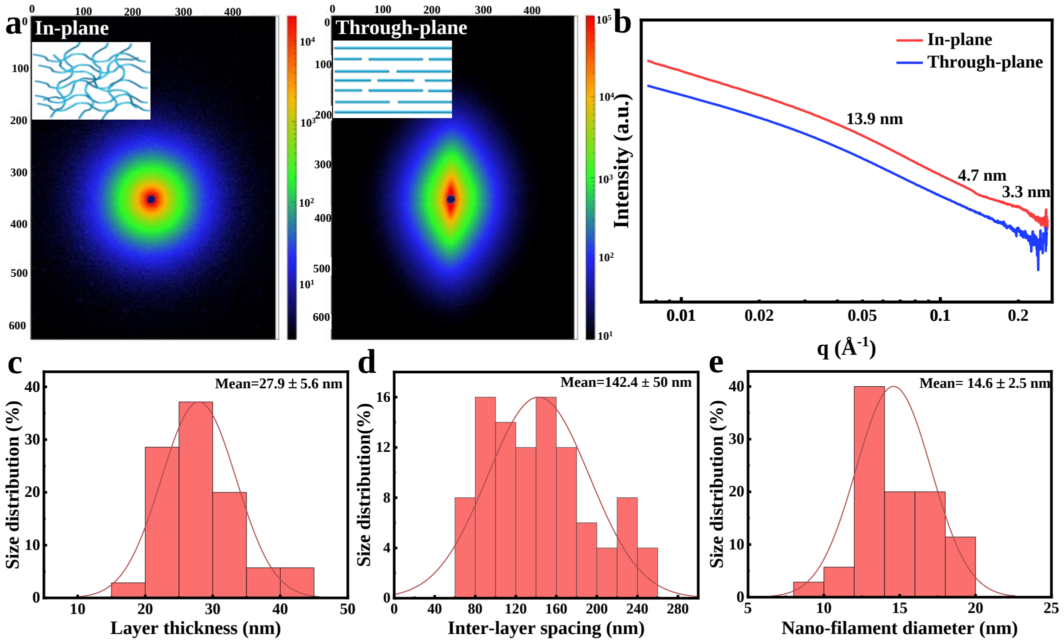

Supplementary Figure 6. Microstructure of gel-state PBI membranes. (a) 2D SAXS images and (b) SAXS patterns of the undoped DC-PBI-G membrane in both in-plane and trans-plane directions. Quantitative analysis includes (c) layer thickness, (d) inter-layer spacing, and (e) diameter of nano-filaments, derived from SEM images.

It's worth noting that sub-nanoscale intermolecular distance, with d-spacing of about 3-10 Å from WAXD analyses, should not be confused with the nano-scale layers observed in SEM images of Fig. 1 f-h. The latter should be the assemblies of lots of the nanoscale molecular layers. Upon the removal of PA and subsequent freeze-drying, gel-state PBI membranes exhibited split layers composed of loosely assembled nano-filaments. The estimated thickness of these layers, derived from SEM images, approximated 27.9 nm, with the inter-layer spacing measuring around 142.4 nm—a dimension beyond the detection capability of WAXD analysis. To further explore the nanometer-level architecture of the membranes, we conducted SAXS analysis, and the findings are detailed in Supplementary Fig. 6. Supplementary Fig. 6b revealed distinct peaks at d-spacings of 13.9 nm, 4.7 nm, and 3.3 nm for DC-PBI-G membrane. These peaks may correspond to the filament bundle sizes of 15 nm or the inter-filament distances.

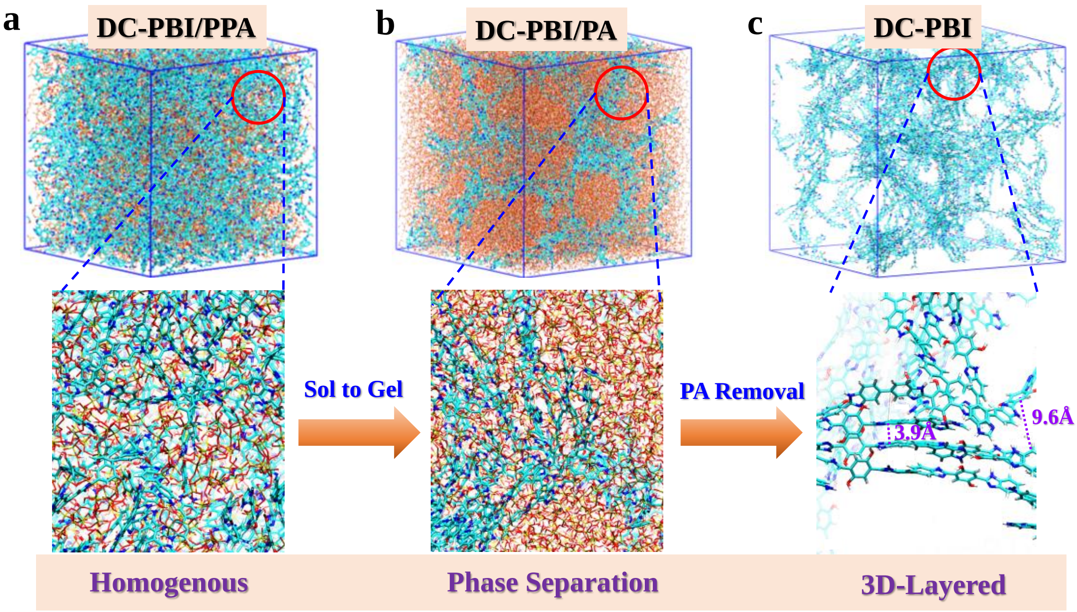

Supplementary Figure 7. MD simulations. 3D spatial configuration and 2D local enlarged views of DC-PBI molecular chains (cubic box size: 12×12×12 nm^3) in (a) PPA, (b) PA, and (c) after PA removal.

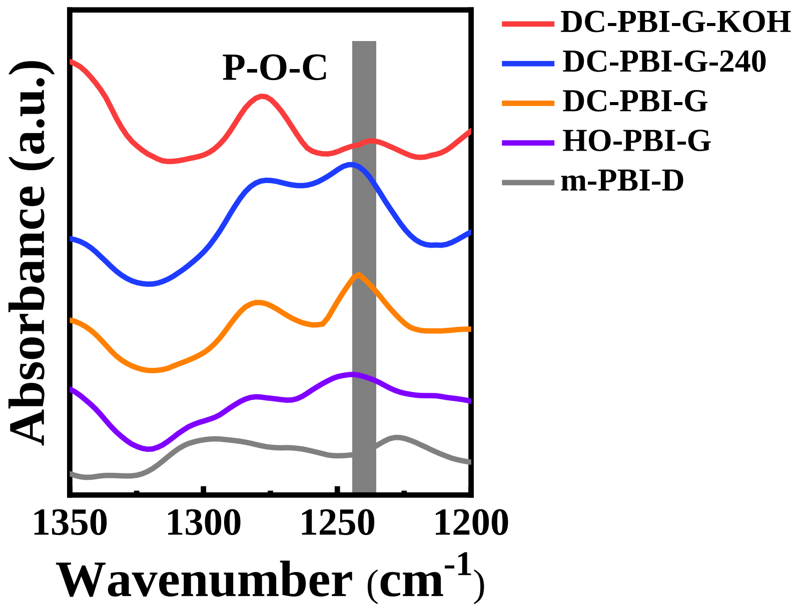

Supplementary Figure 8. FT-IR spectra of various PA-undoped membranes.

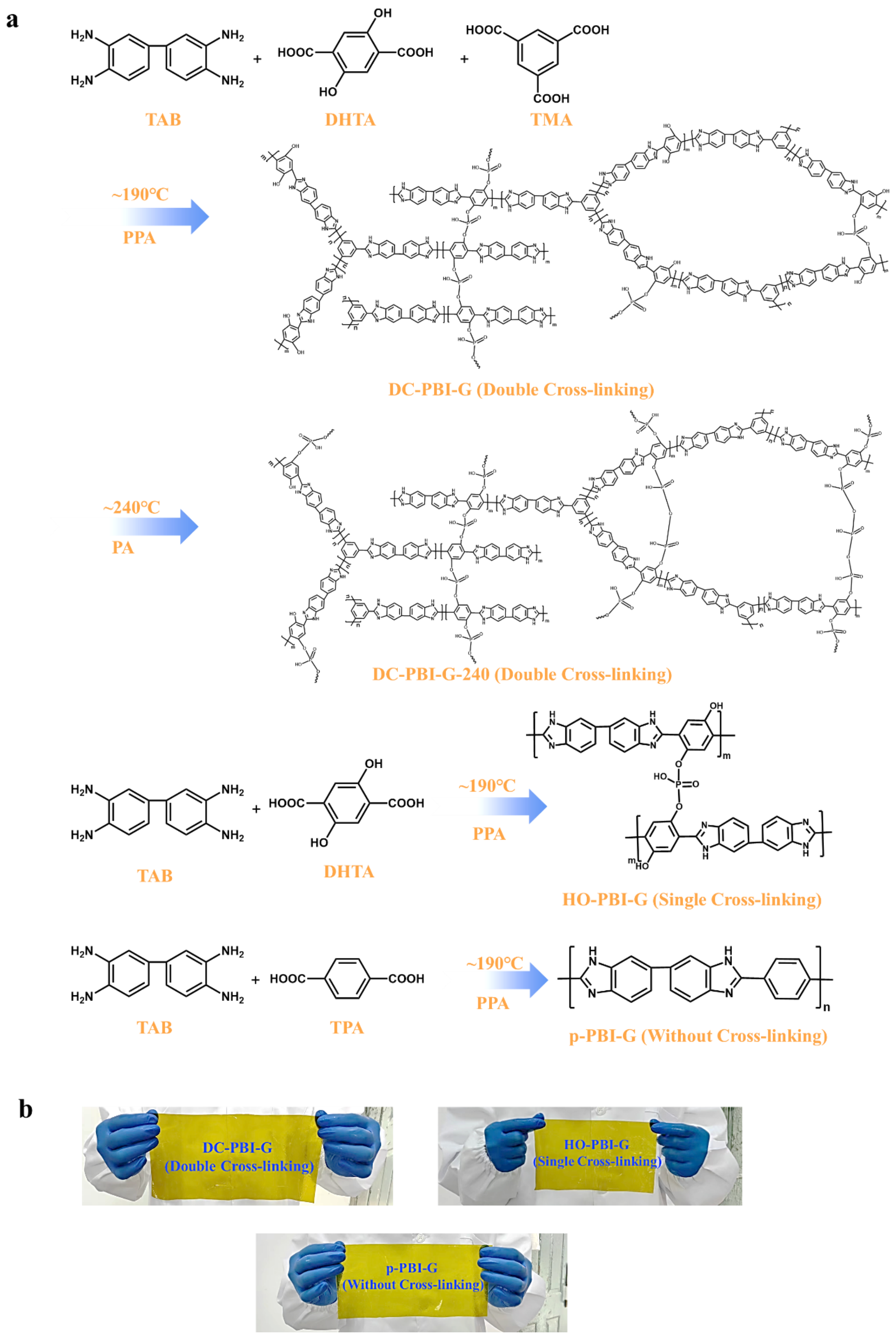

Supplementary Figure 9. Membrane synthesis. (a) Synthetic reactions and (b) photographs of PA-doped DC-PBI-G, DC-PBI-G-240, HO-PBI-G, and p-PBI-G membranes. The manufacturing of membranes was scaled up to about 10 cm × 35 cm.

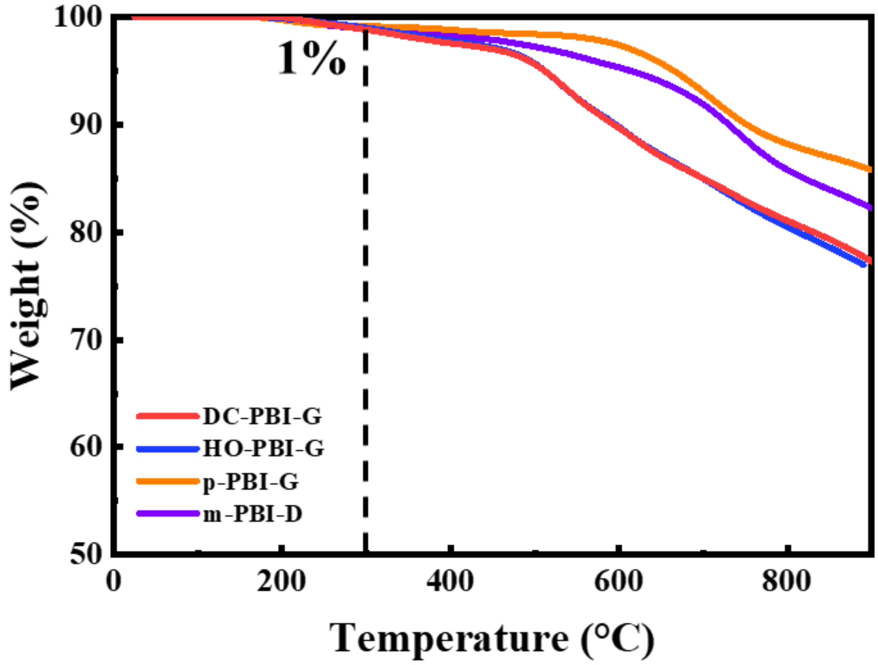

Supplementary Figure 10. TGA curves of PA-undoped PBI membranes.

TGA curves of all membranes in Supplementary Fig. 10 showed initial weight loss around 200 °C for each PBI membrane, causing by the elimination of residual moisture and solvent[4,5]. The subsequent larger weight loss observed around 600 °C corresponded to the decomposition of the main polymer chains. Notably, all membranes exhibited only a 1% weight loss at 300 °C, supporting stable FC operation up to 240 ºC.

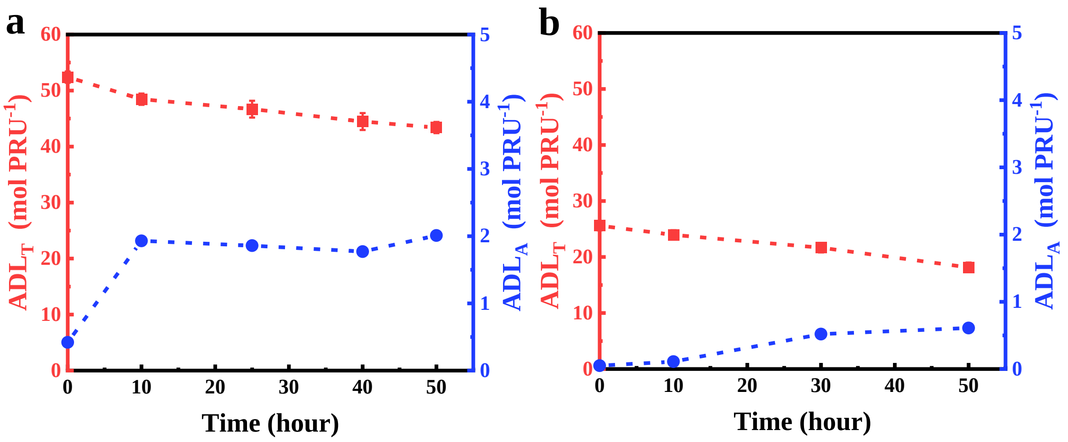

Supplementary Figure 11. Changes in ADL over time at 240 °C. ADL$_T$ and ADL$_A$ for PA-doped (a) DC-PBI-G, (b) p-PBI-G. All error bars indicate the s.d. of at least three independent measurements.

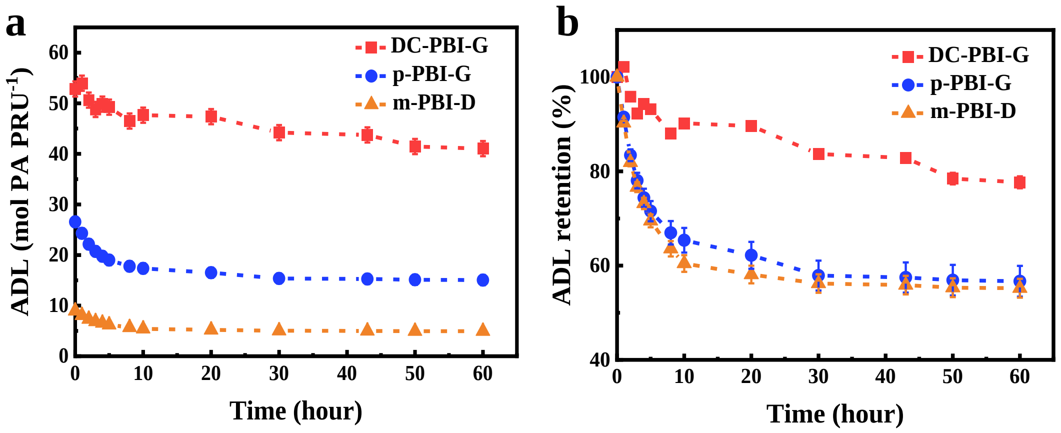

Supplementary Figure 12. PA retention capability at 80 °C/40% RH. (a) The ADL values and (b) retention of PA-doped membranes. All error bars indicate the s.d. of at least three independent measurements.

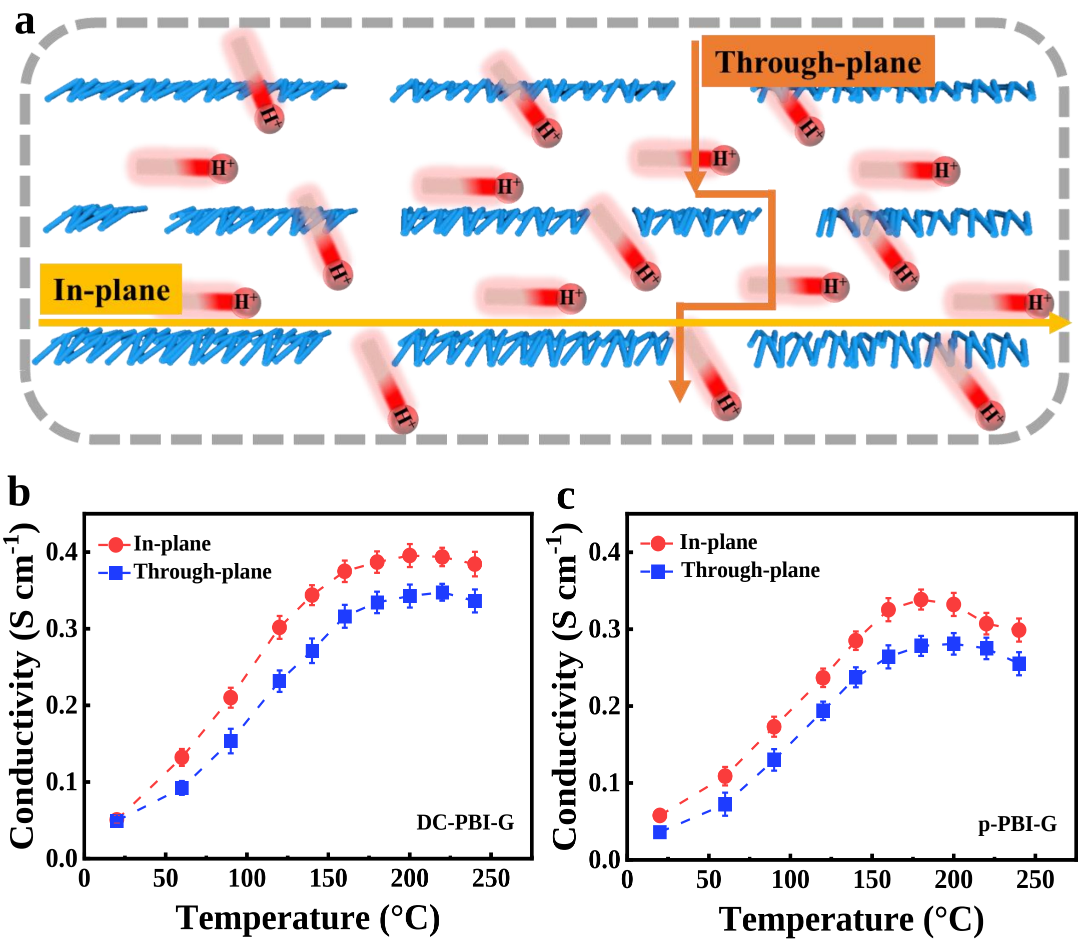

Supplementary Figure 13. Comparative proton conduction in two directions. (a) Schematic diagram of proton transport pathways along the in-plane and through-plane directions. (b) Comparison of in-plane and through-plane proton conductivity for DC-PBI-G. (c) Comparison of in-plane and through-plane proton conductivity for p-PBI-G. All error bars indicate the s.d. of at least three independent measurements.

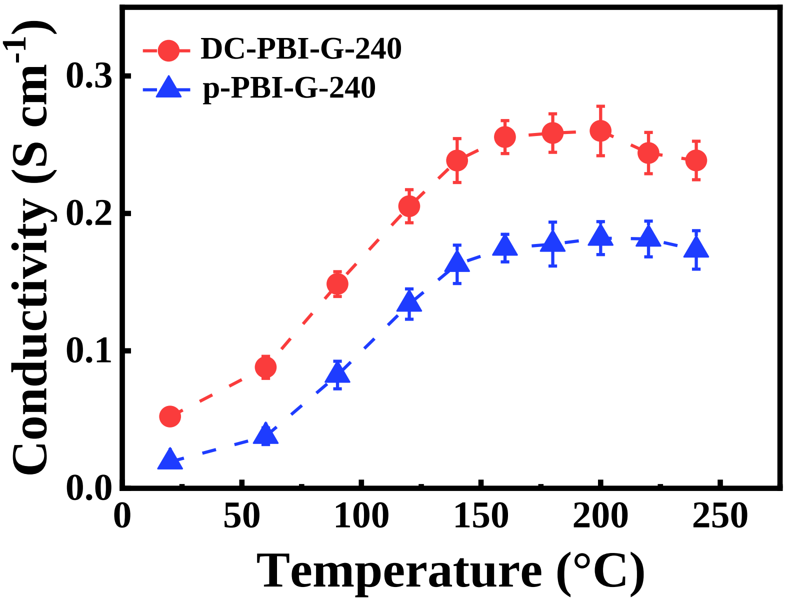

Supplementary Figure 14. Through-plane proton conductivity of PA-doped DC-PBI-G-240 and p-PBI-G-240 membranes at varied temperatures. All error bars indicate the s.d. of at least three independent measurements.

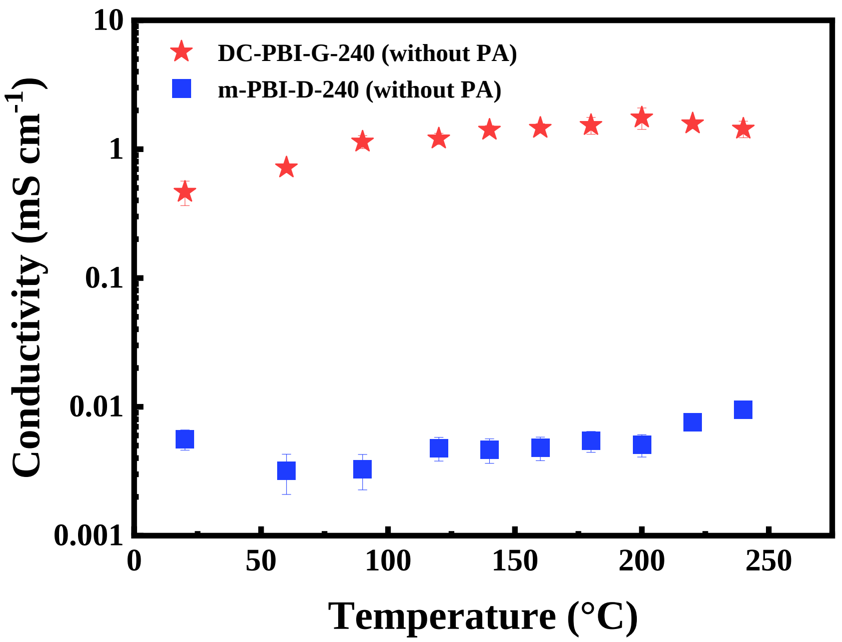

Supplementary Figure 15. Intrinsic through-plane proton conductivity comparison of PA-undoped DC-PBI-G-240 and m-PBI-D-240 membranes. All error bars indicate the s.d. of at least three independent measurements.

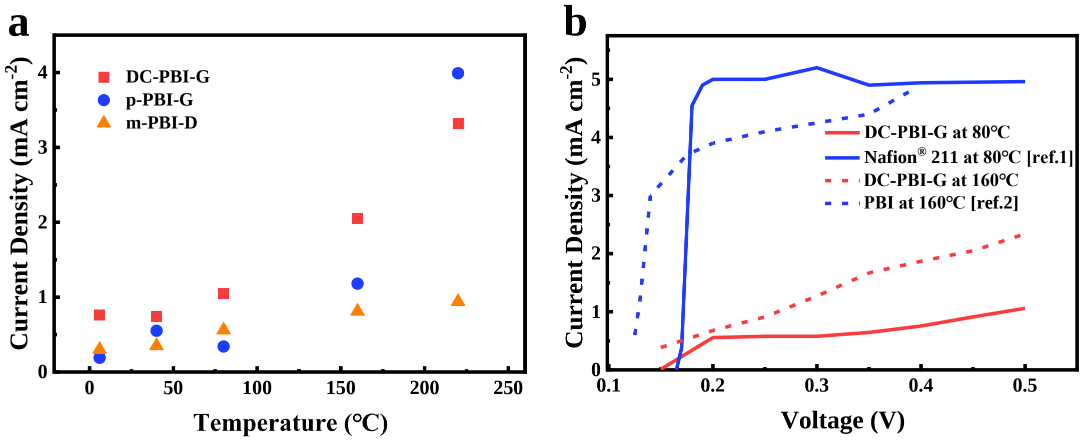

Supplementary Figure 16. H_2 crossover of PA-doped PBI membranes. (a) Equivalent H_2 crossover current densities of the fuel cell based on PA-PBI membrane as a function of operating temperature with ambient pressure at 0.5 V. (b) Linear voltammetry sweeps for Nafion® 211[1] at 80 °C, PBI[2] at 160 °C, and DC-PBI-G at both 80 °C and 160 °C.

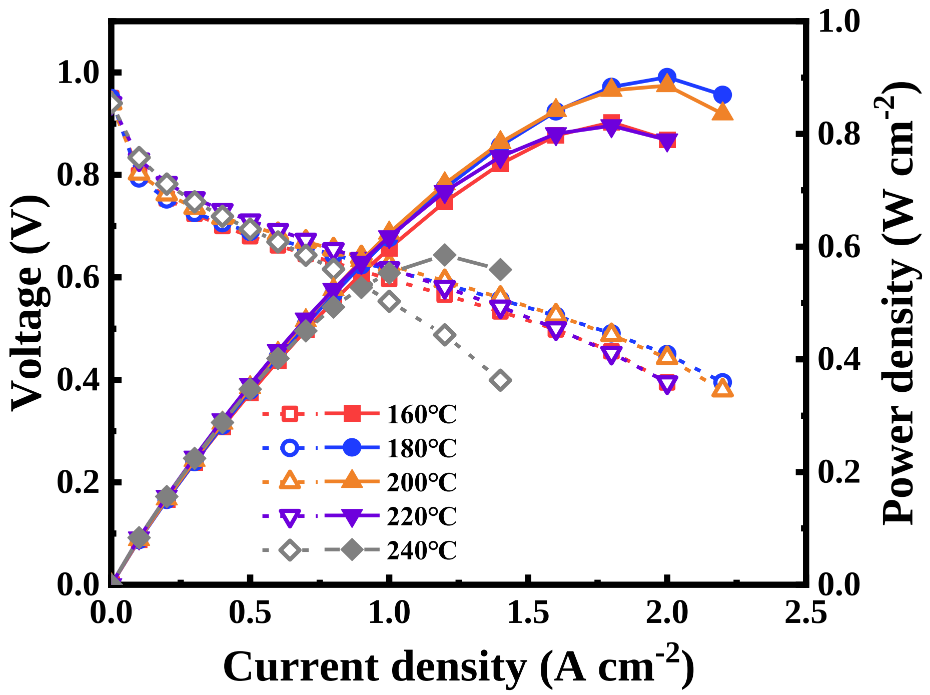

Supplementary Figure 17. Single-cell performance of PA-doped DC-PBI-G under anhydrous conditions with dry H_2/O_2 at atmospheric pressure (Pt loading of 0.5 mg cm^{-2} on both sides).

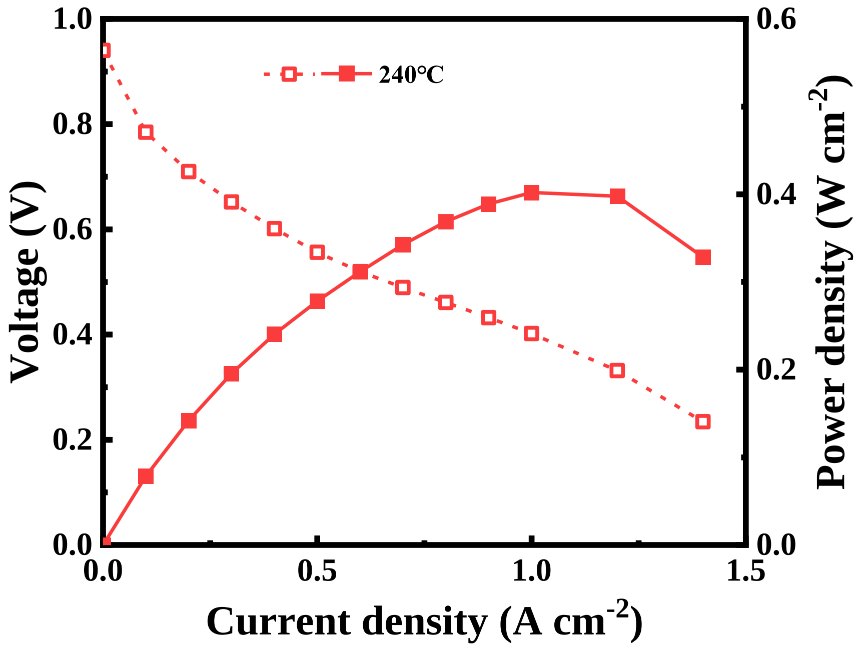

Supplementary Figure 18. Single-cell performance of PA-doped DC-PBI-G-240 under anhydrous conditions with dry H_2/O_2 at atmospheric pressure and 240 °C (Pt loading of 0.5 mg cm^{-2} on both sides).

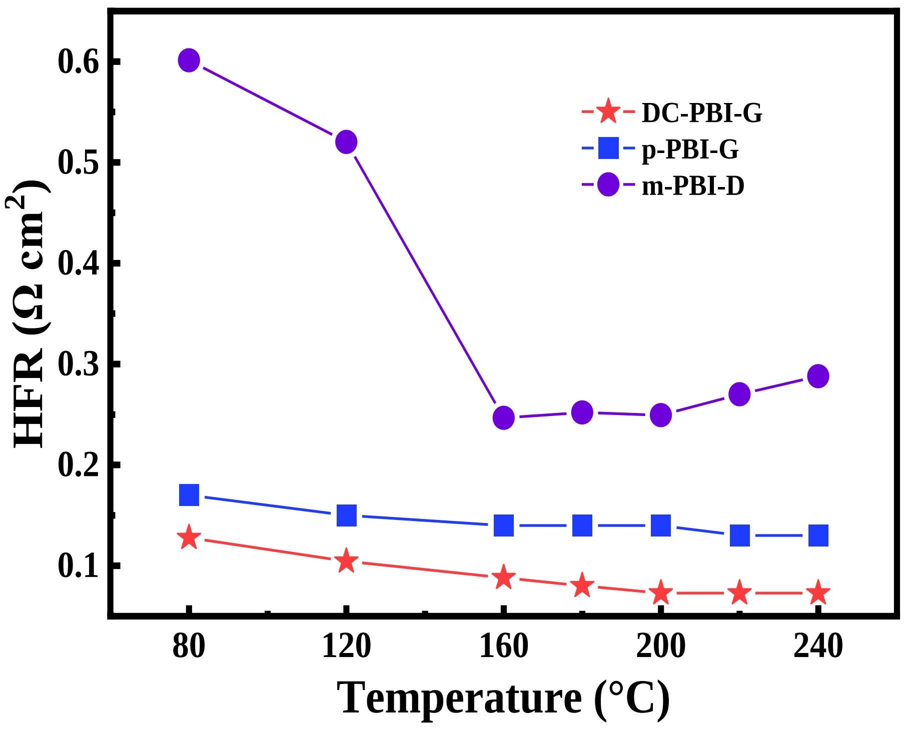

Supplementary Figure 19. High frequency resistance (HFR) of single cell based on PA-doped DC-PBI-G, p-PBI-G and m-PBI-D membranes at varied operating temperatures.

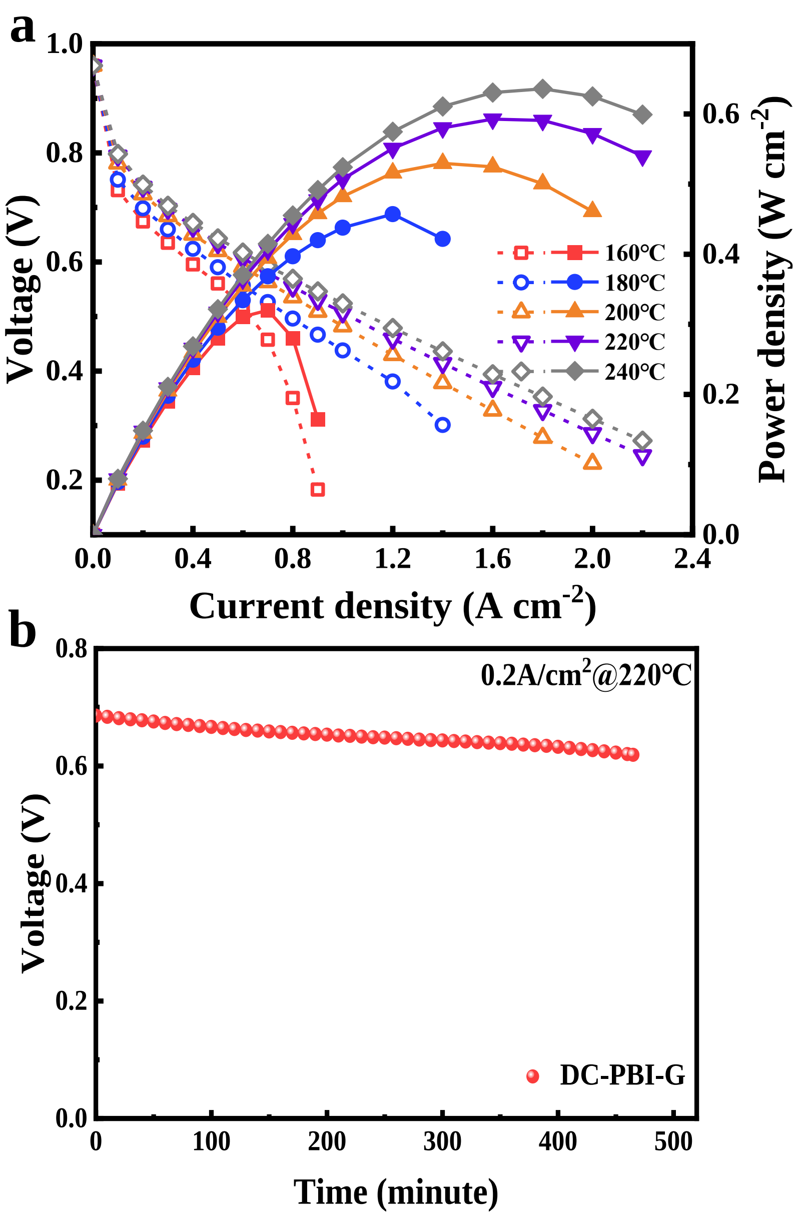

Supplementary Figure 20. Single cell performance of PA-doped DC-PBI-G membrane using dry methanol reformate gas (75% H_2, 24% CO_2 and 1% CO) and O_2 at atmospheric pressure without additional humidification (Pt loading of 1.0 mg cm^{-2} on both sides). (a) Polarization curves and power density at varied operating temperatures. (b) Long-term durability at 0.2 A cm^{-2} and 220 °C.

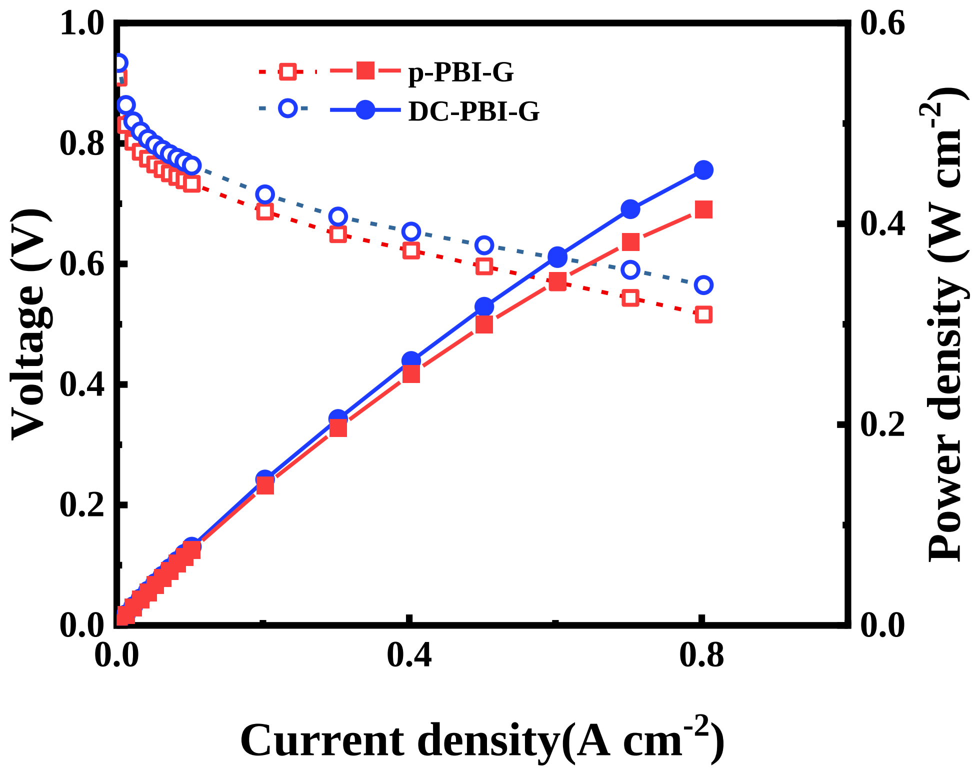

Supplementary Figure 21. Single cell performance of PA-doped p-PBI-G and DC-PBI-G membranes at 160 °C using dry H_2 and air at atmospheric pressure without additional humidification (Pt loading of 1.0 mg cm^{-2} on both sides).

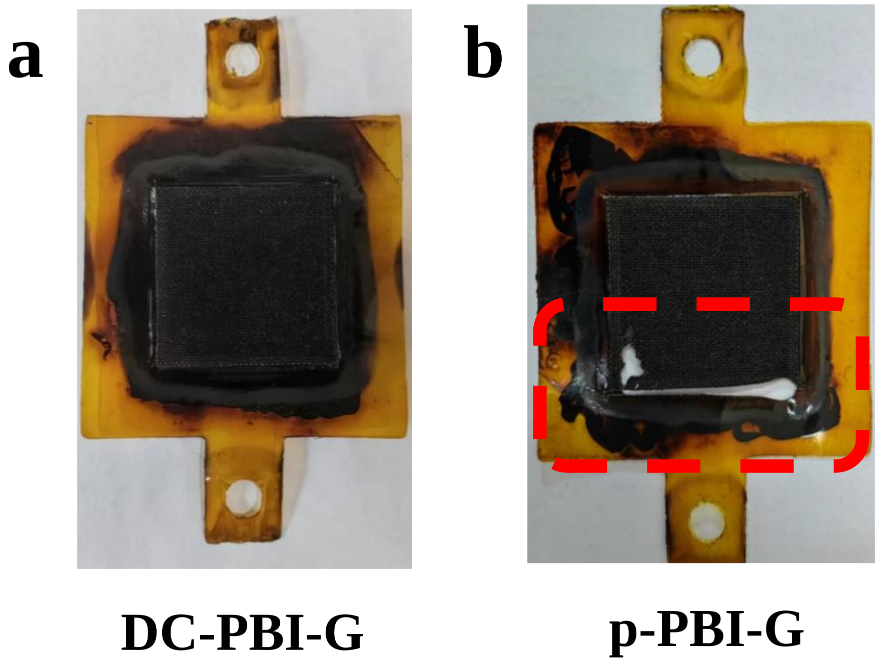

Supplementary Figure 22. Photographs of MEAs. The PA-doped (a) DC-PBI-G and (b) p-PBI-G based MEAs after a long-term durability test at 0.2 A cm^{-2} and 220 °C with dry H_2/O_2 under atmospheric pressure (Pt loading of 1.0 mg cm^{-2} on both sides).

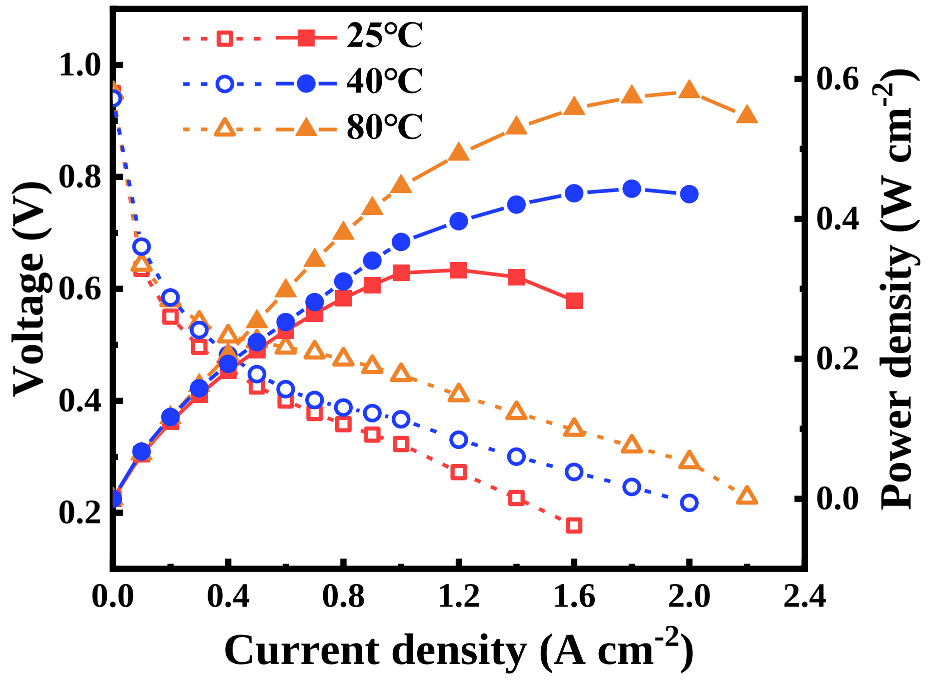

Supplementary Figure 23. Low-temperature single cell performance of PA-doped DC-PBI-G membranes with dry H_2 and O_2 at atmospheric pressure without additional humidification (Pt loading of 1.0 mg cm^{-2} on both sides).

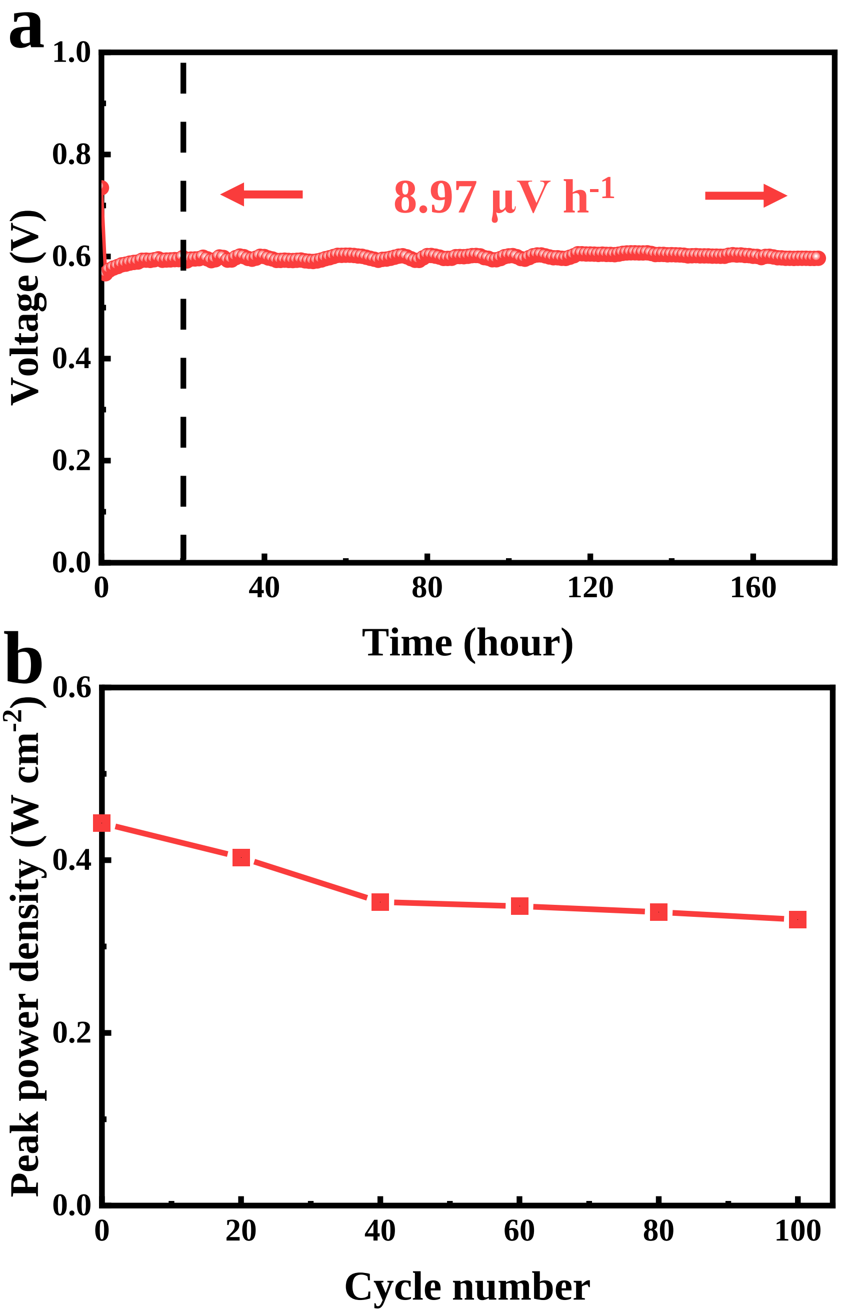

Supplementary Figure 24. Low-temperature FC durability and AST cycling tests. (a) Long-term durability of PA-doped DC-PBI-G MEA with H_2/O_2 at 0.2 A cm^{-2} and 40 °C. (b) Peak power density values of the PA-doped DC-PBI-G MEAs after the shut-down/start-up AST cycling at 40 °C. Testing conditions: Pt loading of 1.0 mg cm^{-2} on both sides, dry H_2/O_2 flow, without backpressure.

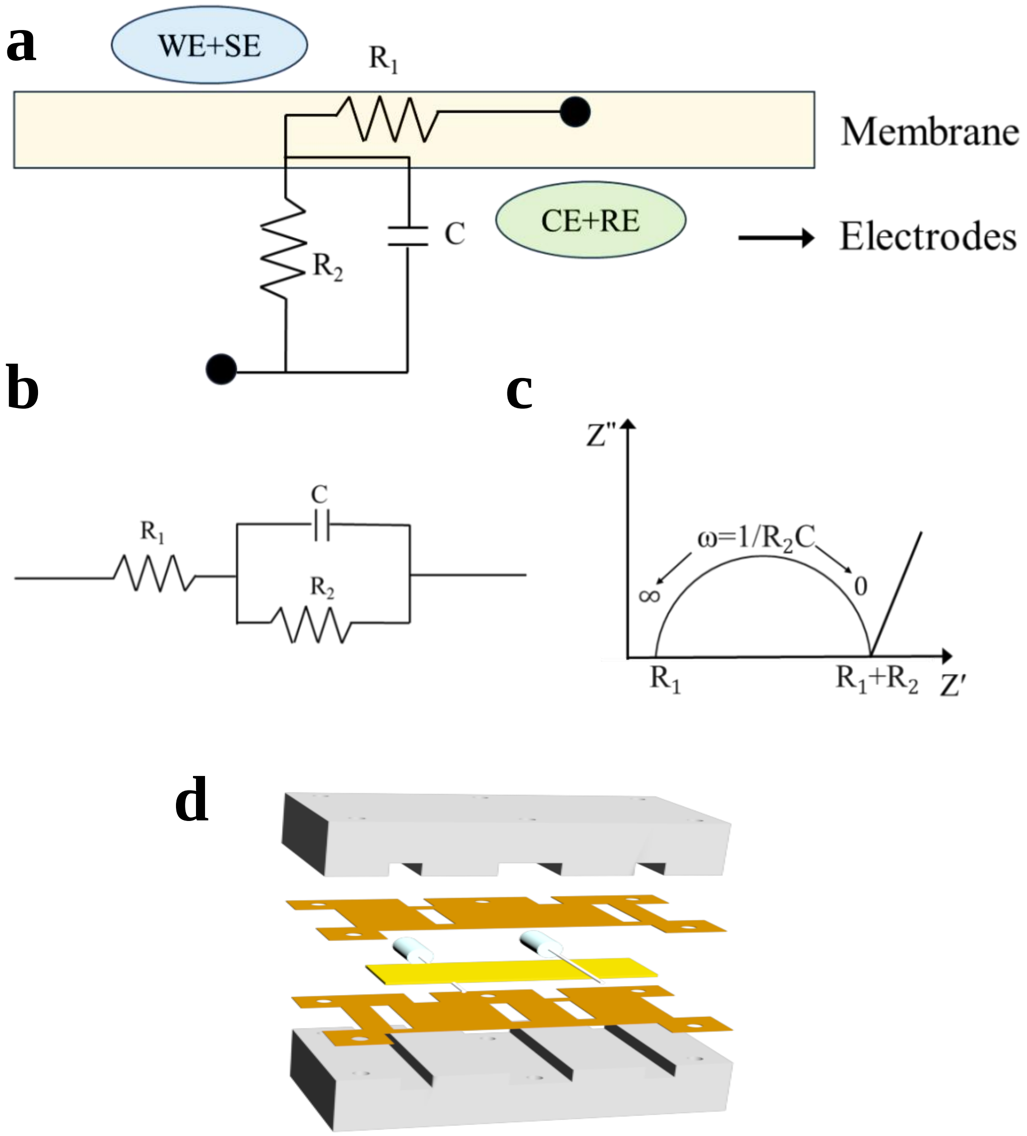

Supplementary Figure 25. Proton conductivity measurement. (a) Schematic of the two-probe conductivity testing setup. (b) Equivalent circuit model. (c) A typical Nyquist plot used for membrane ohmic resistance fitting. (d) Through-plane proton conductivity schematic diagram of the custom-designed impedance test cell utilized in this work.

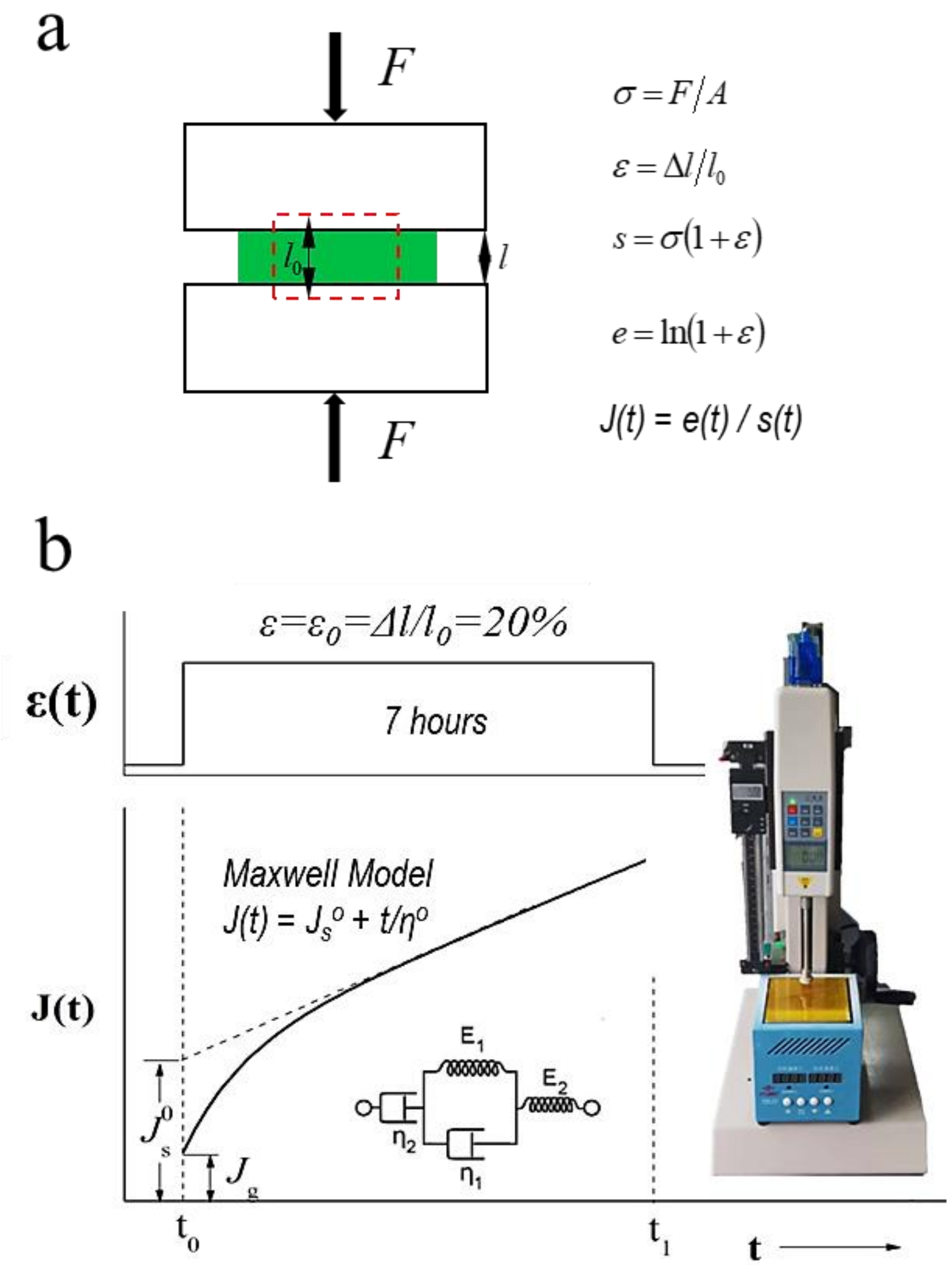

Supplementary Figure 26. Compression creep measurement. (a) Equations for creep compliance estimation. (b) Compression creep testing methodology, Maxwell model and experimental setup.

Supplementary Table 1. Porosity and surface area of undoped PBI membranes.

Membranes	Porosity (%)	Surface area [a] (m^2 g^{-1})	Pore volume [a] (cm^3 g^{-1})
m-PBI-D	18.7 ± 5	1.55	0.019
p-PBI-G	84.6 ± 5	14.78	0.022
**DC-PBI-G**	**95.3 ± 2**	**25.19**	**0.053**

[a] Both surface area and pore volume were assessed through BET analysis. The "±" numbers represent the average of at least three testing samples.

Supplementary Table 2. Comparison of WAXD peak position, area ratio and d-spacing.

		m-PBI-D	p-PBI-G	HO-PBI-G	DC-PBI-G	DC-PBI-G-240
**Peak 1**	2θ (°)	/	10.53	9.94	9.32	8.77
	Area ratio (%)	/	37.6	60.0	44.1	21.4
	d-spacing (Å)	/	8.4	8.9	9.5	10.2
**Peak 2**	2θ (°)	23.01	22.48	25.17	24.46	24.7
	Area ratio (%)	100.0	62.4	40.0	55.9	78.6
	d-spacing (Å)	3.8	3.9	3.5	3.6	3.6

Supplementary Table 3. The elemental mass concentration of PA-undoped DC-PBI-G and p-PBI-G under high-temperature and KOH treatments.

Mass Conc. (%)	C (%)	N (%)	P (%)	P/N Ratio
DC-PBI-G	66.61	6.82	1.55	0.23
DC-PBI-G-240	39.74	1.88	2.02	1.07
DC-PBI-G-KOH	70.52	9.50	0.32	0.06
p-PBI-G	79.22	11.43	0.34	0.03
p-PBI-G-240	66.01	5.13	0.31	0.06
p-PBI-G-KOH	72.70	11.42	0.28	0.02

Supplementary Table 4. The relative integrated peak intensities of ^{31}P NMR spectra.

**Samples**	**P_1**	**P_2**	**P_3**
115PA (PPA)	5	63	32
105PA	53	45	2
85PA	100	0	0
PA-doped p-PBI-G-240	56	2	42
PA-doped m-PBI-D-240	56	42	2
**PA-doped DC-PBI-G-240**	**96**	**2**	**2**

Supplementary Table 5. High-temperature creep compliance and ADL retention of membranes.

**PA-doped membrane**	m-PBI-D	p-PBI-G	HO-PBI-G	**DC-PBI-G**
Creep compliance J^0_s [a] ($10^{-6} Pa^{-1}$)	1.4 ± 0.1	9.7 ± 0.3	5.3 ± 0.2	**2.1 ± 0.1**
ADL retention [b] (%)	75.8 ± 3	83.8 ± 2	89.5 ± 2	**92.6 ± 2**

[a] Creep compliance tests were conducted at 180 °C with a constant compressive strain of 20%. [b] ADL retention was assessed at 240 °C with a constant compressive force equivalent to a stress level of 0.08 MPa. The "±" numbers represent the average of at least three testing samples.

Supplementary Table 6. H_2 crossover current densities and crossover rates of the fuel cell based on PA-PBI membrane as a function of operating temperature with ambient pressure at 0.5 V.

**Equivalent H_2 crossover current densities at indicated temperatures and 0.5 V**						
	6 °C	40 °C	80°C	160 °C	220 °C	**Crossover rates**
DC-PBI-G	0.76 mA cm^{-2}	0.74 mA cm^{-2}	1.05 mA cm^{-2}	2.05 mA cm^{-2}	3.32 mA cm^{-2}	12.2 μA cm^{-2} $°C^{-1}$
p-PBI-G	0.19 mA cm^{-2}	0.55 mA cm^{-2}	0.34 mA cm^{-2}	1.18 mA cm^{-2}	3.99 mA cm^{-2}	15.7 μA cm^{-2} $°C^{-1}$
m-PBI-D	0.30 mA cm^{-2}	0.35 mA cm^{-2}	0.56 mA cm^{-2}	0.81 mA cm^{-2}	0.94 mA cm^{-2}	3.20 μA cm^{-2} $°C^{-1}$

Supplementary Table 7. Summary and comparison of representative LT-PEMs and their corresponding FC performance using H_2.

**HT-PEMs (PA-doped)**	**Conductivity (mS cm^{-1})** @80 °C	**Peak power density (mW cm^{-2})** @80 °C	**Pt loading (mg cm^{-2}) Anode (+), Cathode (-)**	**Reference**
**DC-PBI-G**	**122**	**581**	**1.0**	**This work**
DMBP-TB	~72	~370	0.5 (+)，1.0 (-)	[6]
QAPOH	~104	~130	0.4 (+)，0.6 (-)	[7]
PP-EDTMPA-15/PA	22	325.3	1.0	[8]
PA/1.8TCPP-BrPy-OPBI	71	357	1.0	[9]
p-OPBI-ATMP	~33	268	1.2	[10]
OPBI-CL-Pillar	~56	162	0.6	[11]
OPBI-0.8AM	~38	200	1.0	[12]

BHC2	62	265	1.0	13
porous OPBI	~30	/	0.65	14
BHMP-OPBI	~146	172	0.6	15

Supplementary Table 8. Summary and comparison of representative HT-PEMs and their corresponding FC performance using H_2.

**HT-PEMs (PA-doped)**	**Conductivity (mS cm^{-1})**		**Peak power density (mW cm^{-2})**		**Pt loading (mg cm^{-2})**	**Reference**
	@160 °C	≥200 °C	@160 °C	≥200 °C		
**DC-PBI-G**	**316**	**336 (240 °C)**	**1386**	**1302 (240 °C)**	**1.0**	**This work**
			**821**	**886 (200 °C)**	**0.5**	
p-OPBI-ATMP	112	125 (200 °C)	980	1170 (200 °C)	1.1~1.3	[10]
PBI/$CsH_5(PO_4)_2$	25	26 (200 °C)	450	445 (200 °C)	1.0	[16]
PWA-meso-SiO_2-PBI	19	23 (200 °C)	/	386 (200 °C)	1.6	[17]
PBI/SiO_2	/	41 (200 °C)	/	236 (200 °C)	1.0	[18]
SnP_2O_7–Nafion	80	90 (240 °C)	/	870 (240 °C)	0.6	[19]
PBI/CGP/GO-3	150	/	307	230 (220 °C)	/	[20]

Br-P-40%-OPBI	175	/	1090	/	0.6	[21]
PBI (S-PBI)	230	/	850	/	1.0	[22]
DMBP-TB	150	/	815	/	0.5	[6]
three-layer-OPBI	98	114 (200 °C)	714	/	1.0	[23]
CBOPBI-P40	84	/	699	/	1.0	[24]
50 PPF/PBI	60	/	607	/	1.0	[25]
PIBI-Q80	62	/	600	/	0.5	[26]
OPBI-0.8AM	100	/	560	/	1.0	[12]
30%-CTFsOPBI	72	/	534	/	1.0	[27]
Porous OPBI	71 (180 °C)	/	485	/	0.65	[14]
PA/PVDF-PVP80	72	93 (200 °C)	430	/	0.5	[28]

Supplementary Table 9. Summary and comparison of the FC performance of representative HT-PEMs using reformate gas.

HT-PEMs (PA-doped)	Reformate gas composition				Cathode gas	Peak power density ($mW\ cm^{-2}$)	Pt loading ($mg\ cm^{-2}$)	Reference
	H_2	CO_2	CO	H_2O				
**DC-PBI-G**	**75%**	**24%**	**1%**	**0%**	**Pure O_2**	**636 (240 °C)**	**1.0**	**This work**
PBI/SiO_2	98.7%	0%	1.3%	0%	Pure O_2	210 (200 °C)	1.0	[18]
AB-PBI	98%	0%	2%	0%	Pure O_2	206 (190 °C)	0.5	[29]
Meta-PBI	54%	14%	1%	31%	30% O_2	325 (170 °C)	N/A[a]	[30]
Meta-PBI	75%	22%	3%	0%	Air	266 (160 °C)	1.25	[31]
Meta-PBI	75%	22%	3%	0%	Air	258 (160 °C)	1.0	[32]

[a] Further detail about the catalyst properties cannot be specified due to intellectual property.

References

1 Kutagulla, S. *et al.* Comparative studies of atomically thin proton conductive films to reduce crossover in hydrogen fuel cells. *ACS Appl. Mater. Interfaces* **15**, 59358-59369 (2023).

2 Neyerlin, K. C., Singh, A. & Chu, D. Kinetic characterization of a Pt-Ni/C catalyst with a phosphoric acid doped PBI membrane in a proton exchange membrane fuel cell. *J. Power Sources* **176**, 112-117 (2008).

3 Chen, X., Qian, G., Molleo, M. A., Benicewicz, B. C. & Ploehn, H. J. High temperature creep behavior of phosphoric acid-polybenzimidazole gel membranes. *J. Polym. Sci. Pt. B-Polym. Phys.* **53**, 1527-1538 (2015).

4 Zhu, T. *et al.* Gel-state polybenzimidazole proton exchange membranes with flexible alkyl sulfonic acid side chains for a wider operating temperature range (25-240 °C). *J. Energy Chem.* **85**, 91-101 (2023).

5 Harilal, Nayak, R., Ghosh, P. C. & Jana, T. Cross-linked polybenzimidazole membrane for PEM fuel cells. *ACS Appl. Polym. Mater.* **2**, 3161-3170 (2020).

6 Tang, H. *et al.* Fuel cells with an operational range of-20 ºC to 200 ºC enabled by phosphoric acid-doped intrinsically ultramicroporous membranes. *Nat. Energy* **7**, 153-162 (2022).

7 Lee, K.-S., Spendelow, J. S., Choe, Y.-K., Fujimoto, C. & Kim, Y. S. An operationally flexible fuel cell based on quaternary ammonium-biphosphate ion pairs. *Nat. Energy* **1**, 16120 (2016).

8 Li, W. *et al.* Dual-proton conductor for fuel cells with flexible operational temperature. *Advanced Materials*, 2310584 (2024).

9 Xu, Z. *et al.* Porphyrin helical nanochannel-assembled polybenzimidazole membranes doped with phosphoric acid for fuel cells operating in a temperature range of 25-200 °C. *Adv. Funct. Mater.*, 2310762 (2023).

10 Li, W. *et al.* Porous proton exchange membrane with high stability and low hydrogen permeability realized by dense double skin layers constructed with amino tris (methylene phosphonic acid). *Adv. Funct. Mater.* **33**, 2210036 (2023).

11 Peng, J. *et al.* Achieving over 1,000 mW cm^{-2} power density based on locally high-density cross-linked polybenzimidazole membrane containing pillar[5]arene bearing multiple alkyl bromide as a cross-Linker. *Adv. Funct. Mater.* **33**, 2212464 (2023).

12 Yin, B. *et al.* An effective strategy for the preparation of a wide-temperature-range proton exchange membrane based on polybenzimidazoles and polyacrylamide hydrogels. *J. Mater. Chem. A* **9**, 3605-3615 (2021).

13 Yin, B. *et al.* Construction of stable wide-temperature-range proton exchange membranes by incorporating a carbonized metal-organic frame into polybenzimidazoles and polyacrylamide hydrogels. *Small* **17**, 2103214 (2021).

14 Geng, K., Tang, H. Y., Ju, Q., Qian, H. D. & Li, N. W. Symmetric sponge-like porous polybenzimidazole membrane for high temperature proton exchange membrane fuel cells. *J. Membr. Sci.* **620**, 118981 (2021).

15 Peng, J. *et al.* Constructing novel cross-linked polybenzimidazole network for high-performance high-temperature proton exchange membrane. *J. Membr. Sci.* **643**, 120037 (2022).

16 Li, Y., Hu, J., Li, H. & Chen, L. Performance of an intermediate-temperature fuel cell with a $CsH_5(PO_4)_2$-doped polybenzimidazole membrane. *J. Electrochem. Soc.* **169**, 024505 (2022).

17 Aili, D. *et al.* Exceptional durability enhancement of PA/PBI based polymer electrolyte

membrane fuel cells for high temperature operation at 200 °C. *J. Mater. Chem. A* **4**, 4019-4024 (2016).
18 Cheng, Y. *et al.* High CO tolerance of new SiO_2 doped phosphoric acid/polybenzimidazole polymer electrolyte membrane fuel cells at high temperatures of 200-250 °C. *Int. J. Hydrog. Energy* **43**, 22487-22499 (2018).
19 Lee, K. S. *et al.* Intermediate temperature fuel cells via an ion-pair coordinated polymer electrolyte. *Energy Environ. Sci.* **11**, 979-987 (2018).
20 Singh, B. *et al.* High temperature polymer electrolyte membrane fuel cells with Polybenzimidazole-$Ce_{0.9}Gd_{0.1}P_2O_7$ and polybenzimidazole-$Ce_{0.9}Gd_{0.1}P_2O_7$-graphite oxide composite electrolytes. *J. Power Sources* **401**, 149-157 (2018).
21 Peng, J., Fu, X., Luo, J., Wang, L. & Peng, X. Fabrication of high performance high-temperature proton exchange membranes through constructing stable cation-rich domain in polybenzimidazole membrane. *Chem. Eng. J.* **453**, 139609 (2023).
22 Nam, K. H., Seo, K., Lee, S. & Han, H. Sulfur-doped hierarchically porous open cellular polymer/acid complex electrolyte membranes for efficient water-free proton transport. *ACS Sustain. Chem. Eng.* **8**, 16156-16163 (2020).
23 Wang, P. *et al.* Phosphoric acid-doped polybenzimidazole with a leaf-like three-layer porous structure as a high-temperature proton exchange membrane for fuel cells. *J. Mater. Chem. A* **9**, 26345-26353 (2021).
24 Wang, P., Wu, Y., Lin, W. & Wang, L. Constructing unique carboxylated proton transport channels via the phosphoric acid etching of a metal-organic framework in a crosslinked branched polybenzimidazole. *J. Mater. Chem. A* **10**, 23058-23067 (2022).
25 Hao, X. *et al.* A phosphonated phenol-formaldehyde-based high-temperature proton exchange membrane with intrinsic protonic conductors and proton transport channels. *J. Mater. Chem. A* **10**, 10916-10925 (2022).
26 Ju, Q. *et al.* Performance and stability of ether-free high temperature proton exchange membranes with tunable pendent imidazolium groups. *J. Mater. Chem. A* **10**, 25295-25306 (2022).
27 Peng, J. W. *et al.* Constructing stable continuous proton transport channels by in-situ preparation of covalent triazine-based frameworks in phosphoric acid-doped polybenzimidazole for high-temperature proton exchange membranes. *J. Membr. Sci.* **640**, 119775 (2021).
28 Guo, Z. B., Xu, X., Xiang, Y., Lu, S. F. & Jiang, S. P. New anhydrous proton exchange membranes for high-temperature fuel cells based on PVDF-PVP blended polymers. *J. Mater. Chem. A* **3**, 148-155 (2015).
29 Linares, J. J., Sanches, C., Paganin, V. A. & Gonzalez, E. R. Performance of a poly(2,5-benzimidazole)-based polymer electrolyte membrane fuel cell. *Int. J. Hydrog. Energy* **37**, 7212-7220 (2012).
30 Pinar, F. J., Rastedt, M., Pilinski, N., Wagner, P. & Dyck, A. Demonstrating feasibility of a high temperature polymer electrolyte membrane fuel cell operation with natural gas reformate composition. *Int. J. Hydrog. Energy* **42**, 13860-13875 (2017).
31 Alpaydin, G. U., Devrim, Y. & Colpan, C. O. Performance of an HT-PEMFC having a catalyst with graphene and multiwalled carbon nanotube support. *International Journal of Energy Research* **43**, 3578-3589 (2019).
32 Al-Tememy, M. G. H. & Devrim, Y. Development of effective bimetallic catalyst for high-

temperature PEM fuel cell to improve CO tolerance. *International Journal of Energy Research* **45**, 3343-3357 (2021).
