# Supplementary material for: Double cross-linked 3D layered PBI proton exchange membranes for stable fuel cell performance above 200 °C
Source: Nat Commun. 2024 Apr 22;15:3409. doi: 10.1038/s41467-024-47627-4 (PMC11035571; doi:10.1038/s41467-024-47627-4)
Supplement: Supplementary file 3 — Description of Additional Supplementary Files [file 41467_2024_47627_MOESM3_ESM.pdf]

Description of Additional Supplementary Files

Supplementary Movie 1. The 3D spatial configuration and arrangement of DC-PBI molecular chains (cubic box size: 12×12×12 nm^3) in PPA.

Supplementary Movie 2. The 3D spatial configuration and arrangement of DC-PBI molecular chains (cubic box size: 12×12×12 nm^3) in PA.
